# Impact of Pre-Existing Immunity on Live Attenuated Influenza Vaccine-Induced Cross-Protective Immunity

**DOI:** 10.3390/vaccines8030459

**Published:** 2020-08-20

**Authors:** Sreeja Roy, Clare M Williams, Julian Pardo, Danushka K Wijesundara, Yoichi Furuya

**Affiliations:** 1 Department of Immunology and Microbial Disease, Albany Medical College, Albany, NY 12208-3479, USA; roys3@amc.edu (S.R.); williac10@amc.edu (C.M.W.); 2 Immunotherapy, Inflammation and Cancer, Aragon Health Research Institute, Biomedical Research Centre of Aragon, 50009 Zaragoza, Spain; pardojim@unizar.es; 3 The School of Chemistry and Molecular Biosciences, The Australian Institute for Bioengineering and Nanotechnology, The University of Queensland, Brisbane 4072, Australia; d.wijesundara@uq.edu.au

**Keywords:** influenza, pre-existing immunity, live attenuated influenza vaccine, inactivated influenza vaccine

## Abstract

The efficacy of the intranasally (i.n.) delivered live attenuated influenza vaccine (LAIV) is variable and, in some seasons, suboptimal. In this study, we report that LAIV exhibits cross-protective efficacy in mice, potentially associated with cellular immunity as opposed to antigen-specific antibody responses. However, pre-exposure to the intramuscularly (i.m.) delivered inactivated influenza vaccine (IIV) severely impaired LAIV-induced cross-protection against heterologous challenge, potentially by inhibiting replication of LAIV. Our findings suggest that pre-existing immunity afforded by IIV suppresses cross-protective T cell immunogenicity of LAIV.

## 1. Introduction

The current quadrivalent influenza vaccines contain antigens derived from four influenza viruses of subtypes A and B. These vaccines are mainly either inactivated influenza vaccines (IIVs) (including subunit or split-virion), live attenuated influenza vaccines (LAIVs) or recombinant hemagglutinin (HA) vaccines. Compared to IIV, which predominantly induces strain-specific neutralizing antibody responses, LAIV elicits broad immune responses, including cellular as well as humoral immunity. In experimental animal challenge models of influenza, LAIV consistently elicited superior protective immune responses compared to IIV [1,2], including cross-protective immunity [3]. However, the use of LAIV in infants has been reported to cause wheezing and increased hospitalizations, resulting in recommendation against the use of LAIV in children below the age of 2 years [4]. Therefore, according to the Centers for Disease Control and Prevention’s (CDC) recommended influenza vaccination schedule, most children (age < 2) in the U.S. are exposed to IIV via parenteral route. LAIVs, which are available for individuals 2–49 years of age, will likely be administered to individuals who have previously been vaccinated with parenteral IIV. LAIV, or “flumist”, was withdrawn from the market during 2016–2017 and 2017–2018 influenza seasons because a meta-analysis of combined observational studies from 2013 to 2016 reported reduced effectiveness of LAIV against H1N1 influenza virus [5,6,7]. The clinical report on inferior efficacy of LAIV compared to IIV was in striking contrast to those findings made in animal models across several studies where LAIV was shown to be superior [1,2,3,5]. The discrepancies in LAIV efficacy between animal models and humans raise a question about the predictive value of animal models used for influenza vaccine research, especially if the vaccination context between animal studies and the clinic vary significantly.

Numerous animal and human studies have assessed the role of pre-existing immunity to influenza on subsequent influenza vaccine efficacy [8,9], specifically, the impact of pre-existing immunity on subsequent IIV vaccination. For example, Kim et al. demonstrated that prior influenza infection can enhance the efficacy of subsequent split IIV against drifted strains [9]. However, little is known about the impact of prior vaccination with IIV on LAIV efficacy. Using an infection mouse model, Bodewes et al. showed that prior IIV-derived immunity limits replication of an incoming virus, mitigating protective influenza-specific memory Interferon-gamma (IFN-γ)^+^ CD8^+^ T cell responses induced by heterologous influenza infection [10]. Nevertheless, in animal models, the impact of prior exposure to influenza antigens on LAIV efficacy is often not tested. Thus, we established a mouse model wherein mice were sequentially vaccinated with IIV vaccine followed by LAIV to evaluate if pre-existing immunity can detrimentally impact the efficacy of LAIV. Our sequential vaccinations/infection model shows that pre-existing immunity established by the IIV vaccination suppresses cross-protective efficacy of LAIV, a finding that has important implications for evaluating experimental universal influenza vaccines in animal models. This work may help design better mouse models of influenza to increase the predictive value for vaccine evaluation.

## 2. Materials and Methods 

### 2.1. Immunisation and Challenge

**Figure 1:**
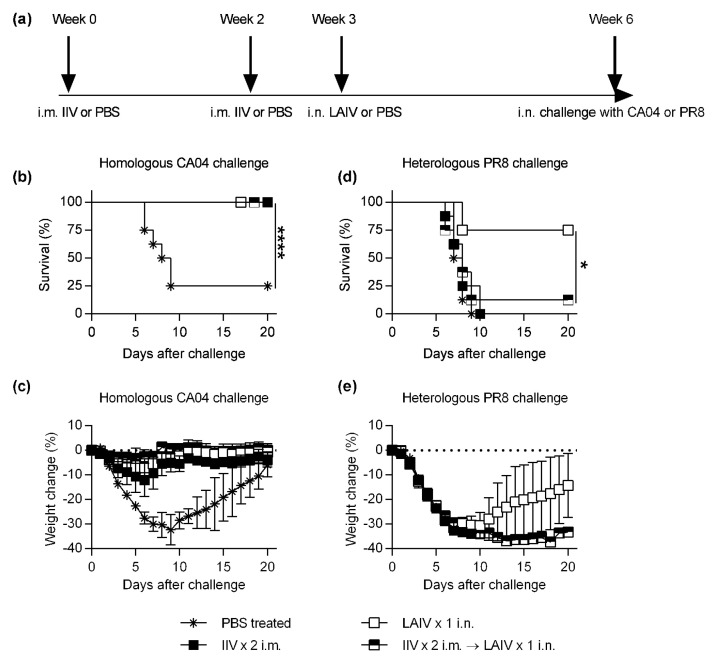
Evaluation of impact of prior IIV vaccination on efficacy of subsequent LAIV following homologous and heterologous influenza challenge. (**a**) BALB/c mice (*n* = 8 per group) were either i.m. vaccinated with IIV or PBS once on week 0 or twice on weeks 0 and 2. Mice were i.n. vaccinated with LAIV or PBS on week 3. Vaccinated mice were i.n. challenged with either homologous CA04 or heterologous PR8 influenza viruses on week 6. Unvaccinated or vaccinated BALB/c mice challenged with CA04 virus were monitored for 20 days for survival and weight loss as described in Section 2. Graphs indicate (**b**) percentage survival and (**c**) percentage of weight change over time. Unvaccinated or vaccinated mice were challenged with PR8 virus and monitored for 20 days for survival and weight loss as described in materials and methods. Graphs indicate (**d**) percentage survival and (**e**) percentage of weight change over time. * *p* < 0.05, **** *p* < 0.0001.

Seven–eight-week-old BALB/c mice were purchased from Charles River Laboratories. Due to the lack of expression of functional M × 1 protein, in-bred laboratory generated BALB/c mice are susceptible to influenza infection. Consequently, these mice have been commonly used for evaluation of influenza vaccine efficacy [11]. Mice were maintained in the Animal Research Facility at Albany Medical College, and all procedures were approved by the Institutional Animal Care and Use Committee. Mice were immunized as indicated in Figure 1a with either 1.5 µg of IIV Fluzone quadrivalent vaccine (2015–2016 formulation, Sanofi Pasteur, Lyon, France) intramuscularly (i.m.) and/or 5 × 10^7^ plaque forming units (PFU) of LAIV Flumist quadrivalent 2015–2016 vaccine (2015–2016 formulation, MedImmune, MA, USA) intranasally (i.n.). Mice were challenged i.n. with either 5 × 10^3^ PFU of homologous H1N1 CA/04/2009 (CA04) virus or heterologous H1N1 A/PR/8/1934 (PR8) virus. Following challenge, mice were monitored for survival and weight loss for 20 days without anesthesia.

### 2.2. Hemagglutination Inhibition (HI) Assay

Serially diluted mouse serum was mixed with either 4 HA units of CA04 virus or 4 HA units of PR8 virus in V bottom 96-well plates. After 30 min incubation, 0.5% of chicken red blood cells (Lampire Biological Laboratories, Pipersville, PA, USA) were added and incubated for an additional 1 h at room temperature. The HI titer was defined as the reciprocal of the highest dilution that prevented viral hemagglutination activity.

### 2.3. Statistical Analysis

GraphPad Prism 7 software was used for statistical analyses. Survival data were analyzed by the Mantel–Cox log-rank test. HI titers were analyzed by either a paired *t* test or two-way ANOVA followed by Sidak’s multiple comparison test. *p* values of <0.05 were considered to be statistically significant. 

## 3. Results

### 3.1. IIV-Derived Pre-Existing Immunity Inhibits LAIV-Induced Cross-Protection

To investigate the impact of pre-existing immunity on LAIV efficacy, mice were first i.m. vaccinated with IIV or PBS and then i.n. immunized with LAIV or PBS (Figure 1a). Following vaccination, mice were challenged with homologous CA04 and monitored for survival and weight loss for 20 days. The mock vaccinated mice showed rapid decrease in survival (survival percentage 25%) and a maximum weight loss by day 9 post challenge (Figure 1b,c). In contrast, mice vaccinated with either IIV or LAIV alone, or a combination of IIV and LAIV, showed 100% survival and prevented significant weight loss following homologous challenge with the CA04 virus (Figure 1b,c). 

Next, we investigated the impact of prior i.m. IIV vaccination on cross-reactive protection elicited following LAIV vaccination. For this purpose, mice were infected with a heterologous PR8 virus following vaccination as indicated in Figure 1a. Data revealed high mortality and morbidity in mice vaccinated with i.m. IIV alone. IIV-vaccinated mice succumbed to PR8 challenge by day 10 (Figure 1d,e). In contrast, nearly all LAIV-only-vaccinated mice survived heterologous PR8 challenge. Although significant weight loss was observed in all the mice, LAIV-vaccinated mice started to recover after day 10 post-infection (Figure 1d,e). However, i.n. LAIV vaccination in IIV pre-vaccinated mice resulted in significantly reduced cross-protection against heterologous PR8 challenge compared to the i.n. LAIV-only vaccination (Figure 1d,e). 

### 3.2. IIV Does Not Impact Subsequent LAIV-Elicited Cross-Protective Hemagglutinin Inhibition (HI) Titers

**Figure 2:**
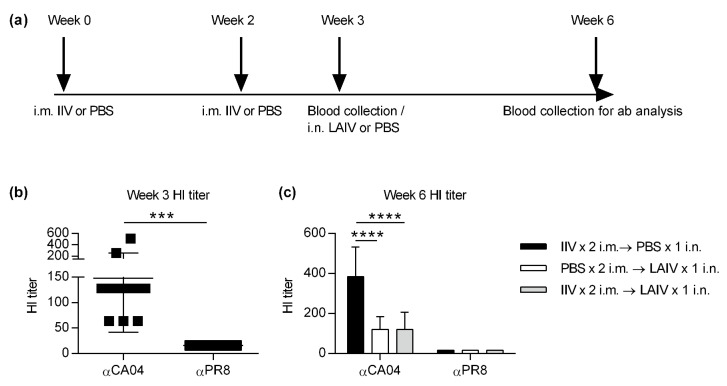
Evaluating the impact of prior IIV on LAIV-induced homologous and heterologous virus-specific antibody titers. (**a**) BALB/c mice were either i.m. PBS treated (control) or vaccinated with each vaccine (i.m. IIV or i.n. LAIV) alone or in combination. Serum harvested on either (**b**) week 3 (*n* = 16 per group) or (**c**) week 6 (*n* = 4–8 per group) as indicated was analyzed for anti-CA04 or anti-PR8 haemagglutinin inhibition (HI titers) as described in Section 2. Error bars represent standard deviation (SD), and *p* values were calculated using paired *t* test (left) and two-way ANOVA followed by Sidak’s multiple comparison test (right). *** *p* < 0.001, **** *p* < 0.0001.

Next, to assess the impact of prior IIV vaccination on LAIV-induced influenza-specific cross-protective antibody responses, cross-reactive serum antibodies were measured by HI assay. Mice were immunized with i.m. IIV, i.n. LAIV or combination of both as described in Figure 2a. Anti-CA04 and Anti-PR8 HI titers were measured 3 weeks after the last vaccination. Data revealed that regardless of vaccination regimen, all vaccinated mice induced significant HI titer against the homologous strain CA04 (Figure 2b,c). In contrast, HI titers against the heterologous PR8 strain were not detected (Figure 2b,c). Thus, while LAIV is capable of providing cross-protection against PR8 virus, it does not induce PR8 specific HI titers. 

## 4. Discussion

The current study demonstrates that prior IIV vaccination does not negatively impact strain-specific immunity induced by LAIV. However, prior IIV vaccination had a detrimental impact on cross-protective immunity elicited by LAIV against heterologous PR8 influenza infection. We speculate that prior IIV vaccination suppresses T cell immunogenicity of LAIV since the observed cross-protection in LAIV-only-vaccinated mice did not correlate with HI titers, indicating an important role of cell-mediated immunity in providing cross-protection. This is unsurprising since numerous mouse studies, including ours have shown the critical role of T cells in mediating cross-protection against influenza [12]. Moreover, LAIV has also been shown to afford cross-protection via cell-mediated immunity [13,14]. Therefore, it is likely that the observed cross-protection in LAIV-only-vaccinated mice is due to the cross-reactive T cells and that prior vaccination with IIV interferes with establishment of LAIV-induced T cell immunity.

One of the major reasons speculated for suboptimal LAIV vaccine efficacy against H1N1 strains is the reduced replicative capacity of vaccine H1N1 pdm09-like strains (A/California/7/2009 for 2013–2014 and A/Bolivia/559/2013 for 2015–2016) in human nasal cells. Replacement of the antigen formulation of LAIV in a subsequent influenza season with H1N1 pdm09-like virus A/Slovenia/2903/2015, which has a better replicative fitness, has indeed improved LAIV-elicited vaccine immunity [15]. Thus, we speculate that the reduced efficacy of LAIV in our mouse model is due to the pre-existing neutralizing antibodies interfering with LAIV replication. Consistent with our hypothesis, a recent phase II clinical trial of a Russian backbone LAIV in Bangladesh has reported that the magnitude of LAIV-induced humoral responses were associated with detectable vaccine virus shedding in vaccine recipients [8]. It was also found that the vaccine recipients with lower pre-existing immunity had higher odds of viral shedding after vaccination. Thus, the vaccine viral infectivity is likely to be a key determinant of vaccine effectiveness. This is a particularly important factor for T cell immunity as in vivo antigen generation and antigen presentation via major histocompatibility complex (MHC)-I is required to prime cytotoxic T cells. Studies have evidenced that prior vaccination suppresses memory IFN-γ^+^ CD8 T cell responses induced by subsequent viral infection [10]. Therefore, taken together, our studies suggest that the pre-existing immunity, which was established by the prior i.m. IIV vaccination, reduced LAIV T cell immunogenicity by limiting LAIV viral replication. Our finding that the pre-existing immunity negatively impacts cross-protective immunity against heterologous PR8, but not strain-specific immunity, has implications for universal flu vaccine development. First, the cross-protective efficacy of an experimental universal flu vaccine should be evaluated in the presence of pre-existing immunity for influenza in animal models, as most humans do have some level of immunity against influenza through seasonal vaccinations and/or exposures to circulating influenza strains. Second, a universal flu vaccine based on live virus or vectors need to consider the infectivity of vaccine strains in the presence of pre-existing immunity. This is particularly a concern for T cell dependent vaccines since pre-existing immunity is more likely to negatively impact T cell, but not humoral, immunogenicity of vaccines. Therefore, third, the mechanism of vaccine mediated cross-protection, which is largely dictated by the vaccine type, becomes critical because pre-existing immunity is likely to have differential impact on humoral vs. cellular immunity. Lastly, the choice of population group needs careful consideration because the magnitude of pre-existing immunity will vary based on age group with very young children expected to have low natural immunity. Thus, in our mouse model, we predict that if LAIV is administered prior to IIV, then cross-protection will be observed. 

## 5. Conclusions

In conclusion, this study highlights that prior IIV vaccination status of a vaccine recipient may be an important factor that determines the efficacy of a universal influenza vaccine. Our data also clearly demonstrate that multiple vaccinations with IIV followed by LAIV result in suboptimal heterosubtypic immunity against heterologous influenza infections.
